# Childrens' and Parents' Willingness to Join a Smartphone-Based Emergency Response Community for Anaphylaxis: Survey

**DOI:** 10.2196/13892

**Published:** 2019-08-27

**Authors:** Michael Khalemsky, David G Schwartz, Tamar Silberg, Anna Khalemsky, Eli Jaffe, Raphael Herbst

**Affiliations:** 1 Graduate School of Business Administration, Bar-Ilan University Ramat Gan Israel; 2 Department of Psychology, Bar-Ilan University Ramat Gan Israel; 3 Edmond and Lily Safra Children’s Hospital, Tel Hashomer Ramat Gan Israel; 4 Israel Magen David Adom Tel Aviv Israel

**Keywords:** mHealth, emergency, volunteer, community, smartphone, anaphylaxis, epinephrine

## Abstract

**Background:**

Medical emergencies such as anaphylaxis may require immediate use of emergency medication. Because of the low adherence of chronic patients (ie, carrying anti-anaphylactic medication) and the potentially long response time of emergency medical services (EMSs), alternative approaches to provide immediate first aid are required. A smartphone-based emergency response community (ERC) was established for patients with allergies to enable members to share their automatic adrenaline injector (AAI) with other patients who do not have their AAI at the onset of anaphylactic symptoms. The community is operated by a national EMS. In the first stage of the trial, children with food allergies and their parents were invited to join.

**Objective:**

This study aimed to identify the factors that influence the willingness to join an ERC for a group of patients at risk of anaphylaxis.

**Methods:**

The willingness to join an ERC was studied from different perspectives: the willingness of children with severe allergies to join an ERC, the willingness of their parents to join an ERC, the willingness of parents to enroll their children in an ERC, and the opinions of parents and children about the minimum age to join an ERC. Several types of independent variables were used: demographics, medical data, adherence, parenting style, and children's autonomy. A convenience sample of children and their parents who attended an annual meeting of a nonprofit organization for patients with food allergies was used.

**Results:**

A total of 96 questionnaires, 73 by parents and 23 by children, were collected. Response rates were approximately 95%. Adherence was high: 22 out of 23 children (96%) and 22 out of 52 parents (42%) had their AAI when asked. Willingness to join the community was high among parents (95%) and among children (78%). Willingness of parents to enroll their children was 49% (36/73). The minimum age to join an ERC was 12.27 years (SD 3.02) in the parents’ opinion and 13.15 years (SD 3.44) in the children’s opinion.

**Conclusions:**

Parents’ willingness to join an ERC was negatively correlated with parents’ age, child’s age, and parents’ adherence. This can be explained by the *free-rider effect*: parents who carried an AAI for their young child, but had low adherence, wanted to join the ERC to get an additional layer of emergency response. Children’s willingness to join the community was positively correlated with age and negatively correlated with the child’s emotional autonomy. Parents’ willingness to enroll their children in an ERC was positively correlated with child’s age and negatively correlated with parents’ adherence: again, this can be explained by the aforementioned *free-rider effect*. Parents’ and children’s opinions about the minimum age to join an ERC were negatively correlated with protective parenting style and positively correlated with monitoring parenting style.

## Introduction

### Objectives of the Study

This study examines different factors affecting willingness of patients and parental-caregivers to join a smartphone-based emergency response community (ERC) for patients with allergy at a risk of anaphylaxis.

### Emergency Response Communities

A medical emergency is “an acute injury or illness that poses an immediate risk to a person’s life or long-term health” [1]. Nontraumatic medical emergencies include conditions such as stroke, severe asthma attack, heart attack, anaphylactic shock, hypoglycemic coma, and drug overdose. The immediate provision of first aid is a crucial factor in lowering mortality and improving long-term prognosis [2,3].

Emergency medical services (EMSs) are the primary provider of first aid to people in medical emergencies that occur outside medical institutions [4,5]. Unfortunately, there is no ambulance on every street corner, and patients in distress have to wait for help. The response times of EMSs vary significantly across countries and geographies, such as rural versus urban areas [6-8].

EMS organizations and health policy makers try to achieve faster response times through various approaches. These include the deployment of automatic electronic defibrillators in public places [9-11], the use of drones to deliver emergency equipment [12], and the establishment of networks of first responders and volunteers to provide first aid in different medical situations [13-18].

An ERC [19,20] is a social network of patients who are prescribed to carry life-saving medication for themselves and can potentially help other patients who are without medication in a medical emergency. Central servers track the location of different community members and locate members who are carrying the required medication and are in the vicinity of the patient in distress. The social network is regulated: the identities and medical records of the patients are verified by their doctors, and the provision of medication in an emergency is approved in real time by EMS personnel. As such, the social network is an *EMS-mediated community of laypersons*.

Joining an ERC requires adoption of a dedicated mobile health (mHealth) smartphone app. mHealth is defined as “healthcare to anyone, anytime and anywhere by removing temporal and locational constraints” [21]. New ERC members have to complete a registration process and agree to location tracking when they are available to respond. The adoption of mobile apps has been widely studied in the past decade—both in general and specifically among patients [22-24].

### Willingness to Join a Mutual Aid Community

ERCs are a type of mutual aid community with members being both potential givers and takers—providers and recipients—of an emergency response. Joining a mutual aid community is a type of volunteerism. Altruism is the most frequently expressed motive for volunteerism. Yet people may volunteer for reasons other than pure altruism. For example, parents may volunteer in an organization from which their children directly benefit [25]. Self-identification as religiously observant is also associated with a higher willingness to join a mutual aid community [26].

The phenomenon of *bystander intervention* has been widely studied over the past 5 decades [27-29]. Mutual aid communities exist in different areas such as among drug addicts [26,30], mental health patients [30,31], and diabetics [32,33].

Another important phenomenon that may influence willingness to join an ERC is *shared identity*: people tend to help those with whom they share something in common [34-36]. As ERC members all share the same medical condition, they may be influenced by this phenomenon—sometimes referred to as *patients like me*.

### Prescription Medication Sharing

There are 2 types of medication sharing: recreational (ie, abusive medication sharing to experience nonmedical effects) and nonrecreational (medication sharing for medical treatment) [37]. ERC is designed to facilitate nonrecreational medication sharing in emergency settings. Prescription medication sharing has been studied for decades [38-40]. A recent meta-analysis, drawing on several studies suggesting that gender, age, income, and use of the internet to access health-related information influence patients’ willingness to share medication [37], reports a wide range of prevalence of the borrowing and lending of prescription medication (5%-52%). Additional studies report the willingness of parents to share or borrow medication related to their child’s medical condition [41]. A recent survey on automatic adrenaline injector (AAI) sharing shows that 76.6% of AAI carriers expressed willingness to share their AAI right away. Respondents who would not share their AAI were concerned about the potential harm to the patient (eg, misdiagnosis or overdose) and to their child (eg, having no medication left or delay in obtaining a refill) [42]. Joining an ERC indicates readiness to share a personal prescription medication with a stranger.

### Parenting Style

To the best of our knowledge, no study has examined the correlation between parents’ willingness to join an ERC and parenting styles associated with medical decision making [43]. Parenting styles are characteristics that represent how parents relate to and place demands on their children [44]. For example, an overprotective parenting style in a family with food allergies is a common coping method that can inhibit a child’s autonomy and lead to the child’s emotional distress over his or her medical condition [45]. The Adult Responses to Children’s Symptoms (ARCS) questionnaire [46] is intended to measure specific health-related parenting practices. The ARCS identifies 3 distinct parenting styles: (1) the protective style, where the parents engage in caretaking behaviors that place the child in a passive patient role, (2) the dismissive style, where the parents criticize the child’s health complaints, and (3) the monitoring style, where the parents encourage the child’s autonomy while monitoring the child’s symptoms [47].

### Child’s Autonomy

Autonomy refers to a person’s ability to act on his or her own values and interest. Taken from ancient Greek, the word autonomy means *self-governance*. From a psychological view, autonomy is made up of a set of functional skills, emotional responses, and attitudes [48]. To act, feel, and reason, the autonomous person must have a sense of self-worth and self-respect.

Some experiences that children and adolescents with food allergies undergo may put them at risk of problems related to the development of their autonomy. Studies show that limiting young children’s opportunities for independent exploration of their environment can interfere with the development of their autonomy [49]. Children with severe food allergies, especially those who have experienced a severe anaphylactic reaction in the past, are likely to be restricted in their activities [50]. On the other hand, adolescents at risk of anaphylaxis are likely to take an active role in managing their allergies [51]. Thus, the findings are mixed: although having restrictions placed on one’s activities is negatively related to the development of autonomy, self-management is positively related to its development.

In this study, we expand the understanding of the sense of autonomy among children with severe allergies. Subsequently, we will refer to autonomy as an agency consisting of 3 components [48]: (1) *attitudinal autonomy*, which refers to the cognitive process of listing one’s possibilities and making a choice between different options; (2) *emotional autonomy*, which refers to conﬁdence and trust in deﬁning goals independent of the wishes of parents and peers; and (3) *functional autonomy*, which describes the process of developing a strategy to achieve one’s goals by means of self-regulation and self-control.

### Description of the field study

EPIMADA is an ERC launched in January 2018 by the Israel National EMS, Magen David Adom (MDA), in cooperation with Bar-Ilan University. EPIMADA comprises patients with allergies who are required to carry an AAI as the first-line treatment against anaphylaxis [52]. Members are equipped with a mobile app that tracks their location and notifies them about relevant emergencies in their vicinity. Members can set preferences such as days of the week and hours of the day during which they are available for dispatch. In an emergency, members are dispatched by the MDA-EMS command center and receive additional real-time instructions from a trained dispatcher-paramedic by phone.

## Methods

We studied the willingness to join an ERC from different points of view:

- Willingness of parents to join an ERC
- Willingness of parents to enroll their children in an ERC
- Willingness of children to join an ERC
- Opinions of parents and children about the minimum age to join an ERC

### Independent Variables

We used several types of independent variables:

- Demographic data (parents’ age, child’s age, parents’ gender, child’s gender, parents’ level of religious observance, and parent’s years of education).
- Medical data about the child (time since diagnosis, time since last anaphylactic attack, and the number of anaphylactic attacks in the past) and medical data about the parents (whether they themselves are allergy patients).
- Adherence (carrying the AAI) both by parents and by children.
- Parenting styles (protective, dismissive, and monitoring).
- Children’s autonomy (attitudinal, emotional, and functional).

### Collection of Data

We used a convenience sample of children diagnosed with severe food allergies and their parents who attended an annual conference of a nonprofit organization of patients with food allergies. All parents were asked to fill out a written questionnaire for parents (Multimedia Appendix 1), and all children older than 8 years were asked to fill out a written questionnaire for children (Multimedia Appendix 2). The questionnaires included a brief description of the EPIMADA project followed by questions about basic parent and child demographics and condition-related factors such as whether the parents are patients themselves, time since diagnosis, adherence level, and emergency events in the past. Parents and children were asked about their willingness to join an ERC; in addition, parents were asked about their willingness to enroll their children. Parents and children were asked for their opinion about the minimum age to join an ERC. Parents completed the adults’ version of ARCS questionnaire, and children completed the children’s version of ARCS questionnaire [46], the Adolescent Autonomy Questionnaire (AAQ) [48], and the Food Allergy Independent Measure (FAIM)[53].

Given that a convenience sample was used, the research is descriptive and does not presume to predict the behavior of the general population.

A total of 23 parent-child pairs attended the conference and answered the parents' and the children's questionnaires (the children had to be at least 8 years old and attend the conference with their parents). All children’s questionnaires were paired with same-family parents’ questionnaires through a coding system that maintained anonymity. A total of 50 parents attended without their children and answered the parents' questionnaires. The response rates were about 95% (2-3 parents arrived too late and were not able to answer the questionnaire because the conference had started. No one refused to answer the questionnaires.). These activities were performed at the start of the conference before participants were informed of the EPIMADA initiative.

### Analytical Techniques

In addition to descriptive statistics, we used several analytical tools to explore our data:

- *t* tests were used, with the assumption of normal distribution, to check if there are significant differences between 2 groups, for example, parents and their children (paired samples) and clusters of parents (independent samples).
- Mann-Whitney nonparametric *U* tests were used as an alternative to *t* tests without the assumption of normal distribution.
- Chi-square independence test was used to check if there is significant association between 2 nominal variables.
- Pearson correlation analysis was used to discover correlations between different variables to plan regression models and to avoid multicollinearity.
- One-way analysis of variances (ANOVAs) were used to check whether there are significant differences between multiple samples, for example, 3 clusters.
- Intraclass correlation (ICC) tests were used to check the consistency of measures between parents and children in the 3 parenting styles.
- Linear regressions were used for scale-dependent variables.
- Ordinal regressions were used for ordinal-dependent variables.
- Binary logistic regressions were used for binary-dependent variables.
- Bootstrapping is a resampling technique that improves the property estimation in small samples. This technique was applied to logistic regressions that initially did not provide significant results.
- Principal component analysis (PCA) was used to find the mix of possibly correlated variables for dimension reduction for cluster analysis.
- Cluster analysis was used in unsupervised learning to enable identification of homogeneous groups without a target attribute by identifying the similarities between objects for a given number of subgroups [54]. This method allows interpretation of the results without relying on an existing target attribute.
- Classification tree (J48) analysis was used in supervised learning with known target variable to enable identification of the most influential variables.

### Institutional Review Board Approval

The research was approved by the Institutional Review Board of Bar-Ilan University and by the Research Committee of MDA.

### Software Tools

The data were converted to digital form by the researchers and analyzed using IBM SPSS 24 software and WEKA 3.7.11 software developed by the University of Waikato in New Zealand.

## Results

### Demographic Parameters of the Sample

A total of 57 (78%) parents were female and 16 (22%) were male. A total of 15 (20.5%) parents reported that they are religiously observant. Tables 1-3 present several demographic parameters of the sample.

**Table 1:** Demographic statistics of parents (n=73).

Parameter	Average	Median	Mode	SD	Min	Max	IQR^a^
Age (N=73)	40.51	40	39	7.15	22	55	9.5 (35.5-45)
Years of education (N=72^b^)	15.74	15.5	15	2.40	12	25	2 (15-17)
Number of children (N=73)	2.49	3	3	0.97	1	6	1 (2-3)

^a^IQR: interquartile range.

^b^These data were missing in 1 questionnaire.

**Table 2:** Age of children.

Parameter	Average	Median	Mode	SD	Min	Max	IQR^a^
Children^b^ (all; N=73)	9.01	8.5	14, 17	5.52	1	21	10 (4-14)
Children^c^ (attended; N=23)	13.69	14	14	3.72	8	21	6 (11-17)

^a^IQR: interquartile range.

^b^Children’s data were provided by all parents about their children.

^c^A total of 23 children attended the conference and filled out children’s questionnaires. The statistics of these children (part of the total sample of 73 children) are based on data reported by their parents. Participation was limited to children aged at least 8 years.

**Table 3:** Gender of children.

Population	Female, n (%)	Male, n (%)
Children (all, N=73)	49 (68)	23 (32)
Children (attended; N=23)	7 (30)	16 (70)

### Medical Statistics

Medical statistical data of children are provided in Table 4.

### Adherence

Parents reported who carries their child’s AAI: in 14 (19%) cases, only the parents carried an AAI; in 19 (26%) cases, only the child carried an AAI; and in 38 (52%) cases, both the parents and the child carried an AAI (in 2 cases, no data were provided).

A total of 52 parents who carried an AAI for their children were asked 3 questions about their own adherence, 57 parents whose children carried an AAI were asked 3 questions about their children’s adherence, and 23 children who attended the conference were asked 2 questions about their adherence. Tables 5-7 present the reports.

We compared the parents’ answers to their children’s answers. A total of 2 children answered “Yes” to the question “Are you carrying an AAI now?” whereas their parents answered “No” to the question “Is your child carrying an AAI now?” When parents and children answered the question “How many days last week did your child have immediate access to an AAI throughout the day?” in 3 cases, parents reported higher adherence (6 vs 4, 7 vs 6, and 7 vs 5) than their children, and in 3 cases parents reported lower adherence than their children (6 vs 7, 5 vs 7, and 1 vs 7).

**Table 4:** Medical statistics of children.

Parameter and N (valid^a,b^)		Average	Median	Mode	SD	Min	Max	IQR^c^
**Time since anaphylaxis diagnosis (years)**								
	62	8.13	7	2	5.59	1	22	9 (3-12)
	20	12.45	12	—^d^	5.36	1	22	7.25 (9.25-16.50)
**Time since last anaphylactic attack (years)**								
	52	4.85	4	1	3.92	1	14	6 (1-7)
	21	6.62	7	1	4.61	1	14	9.5 (1-10.5)
**Number of anaphylactic attacks**								
	70	1.74	1	1	1.77	0	10	2 (1-3)
	22	2.41	2	1	2.15	0	10	2.25 (1-3.25)

^a^Data for all children are reported in the upper row for each variable and data for the children that attended the conference are reported in the lower row.

^b^These data were missing in 1 questionnaire.

^c^IQR: interquartile range.

^d^Multiple modes exist.

**Table 5:** First question about adherence.

Question		Never, n (%)	Seldom, n (%)	Often, n (%)	Always, n (%)	No answer, n (%)
**Reports by parents**						
	Do you make sure to carry the AAI^a^? (N=52)	1 (2)	1 (2)	3 (6)	45 (86)	2 (4)
	Does your child make sure to carry the AAI? (N=57)	2 (4)	0 (0)	10 (18)	43 (75)	2 (3)

^a^AAI: automatic adrenaline injector.

**Table 6:** Second question about adherence.

Question		Yes, n (%)	No, n (%)	No answer, n (%)
**Reports by parents**						
	Are you (parents) carrying an AAI^a^ now? (N=52)	22 (42)	30 (58)	0 (0)
	Is your child carrying an AAI now? (N=57)	53 (93)	3 (5)	1 (2%)
**Reports by chilren**						
	Are you (child) carrying an AAI now? (N=23)	22 (96)	1 (4)	0 (0)

^a^AAI: automatic adrenaline injector.

**Table 7:** Third question about adherence.

Question		1, n (%)	2, n (%)	3, n (%)	4, n (%)	5, n (%)	6, n (%)	7, n (%)
**Reports by parents**						
	How many days last week did you have immediate access to an AAI^a^ throughout the day?^b^ (N=52)	9 (17)	2 (4)	1 (2)	2 (4)	0 (0)	1 (2)	37 (71)
	How many days last week did your child have immediate access to an AAI throughout the day?^b^ (N=57)	1 (2)	0 (0)	0 (0)	1 (2)	1 (2)	3 (5)	51 (89)
**Reports by children**						
	How many days last week did you have immediate access to an AAI throughout the day?^c^ (N=23)	0 (0)	0 (0)	0 (0)	1 (4.3)	1 (4.3)	1 (4.3)	20 (87)

^a^AAI: automatic adrenaline injector.

^b^Reports by parents.

^c^Reports by children.

### Parenting Styles

Parenting styles as assessed by the ARCS questionnaires answered by parents and their children are presented in Table 8.

We performed a paired samples *t* test to compare parenting style assessments based on answers of parents whose children attended the conference with parenting style assessments based on answers of their children who attended the conference. No significant differences were found. A medium positive correlation (*R*=0.451, *P*=.03) was observed between the reports of the parents and their children about the dismissive parenting style. The correlations for other parenting styles were not significant at the 5% significance level. We also performed an ICC test for the 3 parenting styles to check the consistency of measures between parents and children. For the protective parenting style, the ICC value was poor (.319) and not significant (*P*=.06); for the dismissive parenting style, the ICC value was fair (.45) and significant (*P*=.01); and for the monitoring parenting style, the ICC value was poor (.103) and not significant (*P*=.31).

We compared our findings with the data reported by Van Slyke and Walker [47], using a summary independent samples *t* test and found significant differences at the 5% significance level (*P*<.001) in the protective parenting style (Van Slyke and Walker’s results: mean 1.37, SD 0.63) and no significant differences in the dismissive (*P*=.23) and monitoring (*P*=.25) parenting styles.

### Child’s Autonomy

Results related to attitudinal, emotional, and functional autonomy among children who attended the conference are given in Table 9.

**Table 8:** Parenting styles as assessed by the Adult Responses to Children’s Symptoms questionnaires answered by parents and their children.

Parenting style and respondents		Average	Median	Mode	SD	Min	Max	IQR^a^
**Protective**								
	All parents (N=73)	2.39	2.4	2.4	0.69	0.87	3.67	1.06 (1.87-2.93)
	Parents whose children attended (N=23)	2.34	2.33	1.67	0.78	0.87	3.6	1.4 (1.67-3.07)
	Children who attended (N=23)	2.27	2.27	2.93	0.71	1	3.47	1.2 (1.73-2.93)
**Dismissive**								
	All parents (N=73)	0.88	0.83	0.33	0.58	0	2.67	1 (0.33-1.33)
	Parents whose children attended (N=23)	0.74	0.67	0.67	0.62	0	2.17	0.67 (0.33-1)
	Children who attended (N=23)	0.93	0.67	0.5	0.58	0.33	2.17	1 (0.5-1.5)
**Monitoring**								
	All parents (N=73)	2.91	3	—^b^	0.61	0.88	3.88	0.88 (2.5-3.38)
	Parents whose children attended (N=23)	2.94	3.13	3.13	0.61	1.88	3.88	1.12 (2.38-3.5)
	Children who attended (N=23)	2.63	2.63	2.63	0.71	1	3.63	0.87 (2.38-3.25)

^a^IQR: interquartile range.

^b^Multiple modes exist.

**Table 9:** Attitudinal, emotional, and functional autonomy among children who attended the conference (n=23).

Autonomy	Average	Median	Mode	SD	Min	Max	IQR^a^
Attitudinal (N=23)	3.53	3.4	3.4	0.496	2.8	4.4	0.6 (3.2-3.8)
Emotional (N=23)	3.628	3.6	3.8	0.482	2.8	4.4	0.8 (3.2-4)
Functional (N=23)	3.496	3.4	—^b^	0.692	2.4	4.8	1 (3-4)

^a^IQR: interquartile range.

^b^Multiple modes exist.

### Cluster Analysis of Parents

We performed dimension reduction using the PCA technique [55]. We selected the component with the highest percentage of variance explained and selected 10 variables that have correlation of >.4 with the chosen component. We used the *k*-means algorithm with 2 to 4 possible clusters to identify differentiated groups of parents in our sample. We indicate the most prominent attributes that differentiate these groups.

An analysis with 2 clusters of parents identified the 2 groups, which are presented in Table 10. We performed an independent samples *t* test and Mann-Whitney nonparametric *U* tests to check the differences between the parents’ characteristics in these 2 clusters.

The analysis with 3 clusters of parents identified the 3 groups presented in Table 11.

We performed a one-way ANOVA to check the differences between the parents’ characteristics in the 3 clusters. The following differences were significant at the 5% significance level: parent's age (*P*<.001), child’s age (*P*<.001), adherence of parents (*P*<.001), adherence of child (*P*<.001), and willingness to enroll child (*P*<.001), time since the last attack (*P*=.002), and time since diagnosis (*P*<.001).

An analysis with 4 clusters of parents did not reveal any unique cluster with special characteristics. Specifically, the largest group from the 3-cluster analysis was divided into 2 subgroups with slight differences.

### Correlations

Table 12 presents Pearson correlations for scale variables in the parents’ data. A correlation analysis was not performed on the children’s data because of the low number of respondents.

**Table 10:** Parent clusters.

Parameter	Cluster 0	Cluster 1	*t* test (*P* value)	*U* test (*P* value)
Number of cases	41	32	—^a^	—
Parents’ age	36.88	45.16	<.001	<.001
Child’s age	5.66	13.23	<.001	<.001
Children who carry AAI^b^ (valid %)	25 (64)^c^	32 (100)^d^	—	—
Parents who carry AAI for their children (valid %)	38 (97)^c^	14 (44)^d^	—	—
Time since diagnosis (years)	5.53	11.45	<.001	<.001
Time since the last attack (years)	3.82	6.16	.002	.01
Adherence of all parents	2.934	1.79	<.001	—
Adherence of parents who carry AAI for their children	2.97	2.46	<.001	—
Adherence of all children	1.89	2.88	<.001	—
Adherence of children who carry AAI.	2.48	2.88	<.001	—
Adherence: number of days in past week parents had access to AAI^7^	5.89	4.63	.03	.03
Willingness to enroll child in the community	2.04	4.66	<.001	—

^a^Test is irrelevant.

^b^AAI: automatic adrenaline injector.

^c^N=39.

^d^N=32.

**Table 11:** Parent clusters.

Parameter	Cluster 0	Cluster 1	Cluster 2
Number of cases	42	17	14
Parents’ age	39.62	47.23	35.00
Child’s age	8.33	14.85	3.94
Children who carry AAI^a^ (valid %)	40 (100)^b^	17 (100)^c^	0 (0)^d^
Parents who carry AAI for their children (valid %)	37 (92)^b^	1 (6)^c^	14 (100)^d^
Time since diagnosis (years)	7.48	13.18	3.95
Time since the last attack (years)	4.36	6.81	3.92
Adherence of parents	2.80	1.09	2.96
Adherence of parents who carry AAI for their children	2.83	1.00	3.00
Adherence of child (children who carry AAI)	2.63 (2.66)	2.82 (2.82)	0.81 (0.00)
Adherence: number of days in past week parents had access to AAI^7^	5.79	4.22	5.36
Willingness to enroll child in the community	3.07	4.82	1.57

^a^AAI: automatic adrenaline injector.

^b^N=40.

^c^N=17.

^d^N=14.

**Table 12:** Correlations between variables.

Variables^a^	V1	V2	V3	V4	V5	V6	V7	V8	V9	V10	V11	V12	V13
V1	1	0.143	0.291^b^	0.828^c^	0.793^c^	0.098	−0.115	0.029	0.502^c^	0.277^b^	−0.267^b^	0.086	−0.292^b^
V2	0.143	1	0.017	0.039	0.087	−0.248^b^	0.099	0.38	0.204	−0.299^b^	−0.054	−0.280^b^	0.099
V3	0.291^b^	0.017	1	0.326^c^	0.316^b^	0.022	–0.009	−0.215	0.131	0.237^b^	0.052	0.060	0.011
V4	0.828^c^	0.039	0.326^c^	1	0.91^c^	0.163	−0.048	−0.086	0.481^c^	0.455^c^	−0.187	0.202	−0.249^b^
V5	0.793^c^	0.087	0.316^b^	0.91^c^	1	−0.021	−0.091	−0.095	0.529^c^	–0.039	0.26^b^	−0.102	0.487^c^
V6	0.098	−0.248^b^	0.022	0.163	−0.021	1	0.200	−0.091	0.011	−0.012	−0.114	0.034	0.027
V7	−0.155	0.099	−0.009	−0.048	−0.091	0.200	1	0.199	−0.035	−.030	−0.075	0.008	0.008
V8	0.029	0.038	−0.215	−0.086	−0.095	−0.091	0.199	1	−0.056	−0.003	0.082	0.060	0.135
V9	0.502^c^	0.204	0.131	0.481^c^	0.529^c^	0.011	−0.035	−0.056	1	−0.205	−0.240	−0.054	−0.177
V10	0.277^b^	−0.299^b^	0.237^b^	0.455^c^	−0.039	−0.012	−0.030	−0.003	−0.205	1	0.142	0.511^c^	0.013
V11	−0.267^b^	−0.054	0.052	−0.187	0.26^b^	−0.114	−0.075	0.082	−0.240	0.142	1	0.188	0.634^c^
V12	0.086	−0.280^b^	0.060	0.202	−0.102	0.034	0.008	0.060	−0.054	0.511^c^	0.188	1	−0.056
V13	–0.292^b^	0.099	0.011	−0.249^b^	0.487^c^	0.027	0.008	0.135	−0.177	0.013	0.634^c^	−0.056	1

^a^The list of variables used are as follows: V1, parents’ age; V2, parents’ years of education; V3, number of children; V4, child’s age; V5, time as diagnosis (years); V6, parents’ opinion about the minimum age for a child to join an ERC; V7, number of days in past week parent who had access to AAI; V8, number of days in past week child who had access to AAI; V9, time as last anaphylactic attack; V10, number of anaphylactic attacks in the past; V11, protective parenting style; V12, dismissive parenting style; V13, monitoring parenting style.

^b^Significant at the 5% level.

^c^Significant at the 1% level.

### Parents’ Willingness to Join an Emergency Response Community

Parents were asked 2 questions about their willingness to join an ERC. A total of 69 parents (95%) answered “Yes” to the yes-or-no question “Do you intend to join the community?” Because of the very high percentage of affirmative questions, no further statistical analysis (eg, logistic regression) was possible.

Parents were also asked about the probability (0 [very unlikely] to 6 [very likely]) that they would join an ERC. Their answers are presented in Table 13.

We used an ordinal regression model to analyze the factors that influence the probability of joining an ERC. We used the following independent variables: child’s gender, child’s age, parents’ age, parents’ education, parents’ adherence (number of days in past week parent had immediate access to AAI), child’s adherence (number of days in past week child had immediate access to AAI), time since diagnosis, the number of anaphylactic attacks in the past, time since the last attack, and parenting style. The model fitting was significant (χ^2^_13_=22.9 with *P*=.03). The goodness of fit was significant at the 5% significance level for the Pearson chi-square test (*P*<.001). The pseudo *R*^2^ indicators were Cox&Shell=0.449 (44.9% of the total variability explained by the model), Nagelkerke=0.507, and McFadden=0.276. A threshold check showed significant differences between most of the values of the dependent variable at the 5% significance level. The following independent variable estimates were significant at the 5% significance level: parents’ age (an increase in age was associated with a decrease in the probability of joining the community, with an odds ratio [OR] of 0.688 [95% CI 0.49-0.96], Wald χ^2^_1_=4.7, *P*=.03), parents’ adherence (number of days in past week parent had immediate access to AAI); (an increase in parents’ adherence was associated with a decrease in the probability of joining the community, with an OR of 0.586 [95% CI, 0.38 to 0.91], Wald χ^2^_1_=5.7, *P*=.02), child’s adherence (number of days in past week child had immediate access to AAI); (an increase in child’s adherence was associated with an increase in the probability of joining the community, with an OR of 2.106 [95% CI 1.18-3.75], Wald χ^2^_1_=6.4, *P*=.01).

A 2-tailed t test for independent samples assuming equal variances (according to Levin’s test) and a Mann–Whitney nonparametric U test showed no significant differences in the probability of the parent joining the community by parents’ gender, but a 1-tailed t test revealed that females are more likely to join the community than males at the 5% significance level (P=.04).

A 2-tailed t test for independent samples assuming equal variances (according to Levin’s test) and a Mann–Whitney nonparametric U test showed no significant differences in the probability of the parent joining the community by child’s gender.

**Table 13:** Parents’ answers about the probability of them joining the community (N=73).

Answer	n (%)
0 (very unlikely)	1 (1.4)
1	2 (2.7)
2	1 (1.4)
3	5 (6.8)
4	7 (9.6)
5	11 (15.1)
6 (very likely)	45 (61.6)
No answer	1 (1.4)

### Children’s Willingness to Join an Emergency Response Community

Children were asked 2 questions about their willingness to join an ERC. A total of 18 out of 23 children (78%) answered “Yes” to the yes-or-no question “If your parents let you, do you intend to join the community?“

Because of the low number of respondents, a logistic regression was not able to analyze the factors that influence children’s opinions about joining the community.

Children were also asked what the probability was (0 [very unlikely] to 6 [very likely]) that they would join an ERC, assuming that their parents would allow them to join. Their answers are presented in Table 14.

We used an ordinal regression model to analyze the factors that influence the probability of joining an ERC. We used the following independent variables: child’s gender, child’s age, child’s adherence (number of days in past week child had immediate access to AAI), parenting style, and child’s autonomy. The model fitting was not significant. The goodness of fit was significant at the 5% significance level for the Pearson chi-square test (*P*<.001). The pseudo *R*^2^ indicators were Cox&Shell=0.437 (43.7% of total variability explained by the model), Nagelkerke=0.453, and McFadden=0.173. A threshold check resulted in nonsignificant differences between all values of the dependent variable at the 5% significance level. The following independent variables estimates were significant at the 5% significance level: age (an increase in age was associated with an increase in the probability of joining the community, with an OR of 1.508 [95% CI 1.13-2.02], Wald χ^2^_1_=7.6, *P*=.006) and emotional autonomy (an increase in emotional autonomy was associated with a decrease in the probability of joining the community, with an OR of 0.038 [95% CI 0.0018-0.79], Wald χ^2^_1_=4.4, *P*=.04). Because of the low number of respondents, the ability of the model to explain the variability is limited. A series of *t* tests for independent samples assuming equal variances (according to Levin test) and Mann-Whitney nonparametric *U* tests showed no significant differences by child’s gender in the probability of a child joining the community. A chi-square test showed no significant differences between male and female children in the probability of joining the community.

**Table 14:** Children’s answers about the probability of them joining the community (N=23).

Answer	n (%)
0 (very unlikely)	2 (9)
1	1 (4)
2	3 (13)
3	1 (4)
4	9 (39)
5	2 (9)
6 (very likely)	5 (22)
No answer	0 (0)

### Parents’ Willingness to Enroll Their Children in an Emergency Response Community

Parents were asked 2 questions about their willingness to enroll their children in an ERC. A total of 36 parents out of 73 (49%) answered “yes” to the yes-or-no question “Do you intend to enroll your child in the community?”

We used a logistic regression to analyze the factors that influence the parents’ decision to enroll their children in the community (dependent variable). We used the following independent variables: parents’ age, parents’ education, number of children, child’s age, time since diagnosis, parents’ adherence (number of days in past week parents had immediate access to AAI), child’s adherence (number of days in past week child had immediate access to AAI), time since the last anaphylactic attack, the number of anaphylactic attacks in the past, and parenting style. Omnibus tests of model coefficients provided significant results with *P*=.04, Cox&Shell *R*^2^=0.426, and Nagelkerke *R*^2^=0.574. According to the model, all independent variables were included in the equation, but only 1 was significant at the 5% significance level: parents’ adherence (days in the past week parent had immediate access to AAI) was negatively associated with the willingness to enroll the child into the community. Bootstrapping with 1000 iterations provided the following significant variables at the 5% significance level: parents’ age (negative), parents’ education (negative), child’s age (positive), time since diagnosis (positive), the number of anaphylactic attacks in the past (negative), parents’ adherence (number of days in the past week parent had immediate access to AAI; negative), and child’s adherence (number of days in the past week child had immediate access to AAI; positive).

**Figure 1:**
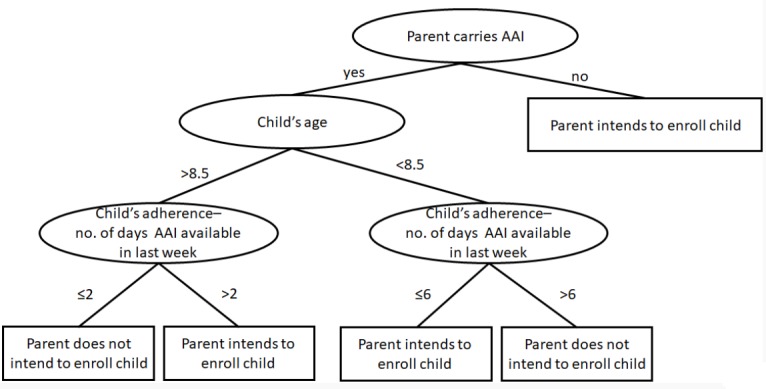
The influence of different independent variables on the parents’ decision to enroll their children in the community.

We performed another analysis of this variable by applying the J48 classification tree to evaluate the influence of different independent variables on the parents’ decision to enroll their children in the community. The tree correctly classifies 71.21% of the cases. Figure 1 presents the results.

In an attempt to expand the options scale, the parents were also asked the question, “What is the probability (0–6) that you will enroll your children in an ERC?” Their answers are presented in Table 15.

We used an ordinal regression model to analyze the factors that influence the probability that parents will enroll their child in an ERC. We used the following independent variables: parents’ age, parents’ education, number of children, child’s age, parents’ adherence (number of days in the past week parent had immediate access to AAI), child’s adherence (number of days in the past week child had immediate access to AAI), time since the last anaphylactic attack, the number of anaphylactic attacks in the past, parenting style, and child’s gender. The model fitting was not significant (χ^2^_13_=19.1 with *P*=.09). The goodness of fit was significant at the 5% significance level for the Pearson chi-square test (*P*=.02). The pseudo *R*^2^ indicators were Cox&Shell=0.359 (35.9% of total variability explained by the model), Nagelkerke=0.372, and McFadden=0.133. A threshold check resulted in insignificant differences among all 7 values of the dependent variable. Bootstrapping did not improve these results.

**Table 15:** Parents’ answers about the probability (0 [very unlikely] to 6 [very likely]) of enrolling their children in the community (N=73).

Answer	n (%)
0 (very unlikely)	19 (26.0)
1	3 (4.1)
2	4 (5.5)
3	5 (6.8)
4	6 (8.2)
5	8 (11.0)
6 (very likely)	19 (26.0)
No answer	9 (12.3)

### Opinions About Minimum Age to Join an Emergency Response Community

Both parents and children were asked their opinions about the minimum age to join an ERC (see Table 16).

In a paired samples *t* test, no significant differences were found between children’s reports and their parents’ reports. In an independent samples *t* test, no significant differences were found between males and females. A series of Pearson correlation tests revealed a weak negative (–.246) correlation between parents’ opinions and parents’ years of education (*P*=.049). No significant correlations of parents’ opinions were found either with parents’ age or with their children’s age.

We used a linear regression to analyze the factors that influence parents’ opinions about the minimum age to join an ERC. We used the following independent variables: parents’ age, parents’ education, number of children, child’s age, parents’ adherence (number of days in the past week parent had immediate access to AAI), child’s adherence (number of days in the past week child had immediate access to AAI), time since the last anaphylactic attack, parenting style, and number of anaphylactic attacks in the past. A multicollinearity analysis did not reveal any evidence of multicollinearity. The model resulted in *R*^2^=0.55, and the model was significant at the 5% significance level (*P*=.004). An analysis of standardized regression coefficients revealed that the most influential significant variables are child’s age (beta=0.489, standardized beta=0.801, *P*=.003), protective parenting style (beta=–2.767, standardized beta=–0.561, *P*=.01), parent's age (beta=–0.242, standardized beta=–0.526, *P*=.03), monitoring parenting style (beta=2.584, standardized beta=0.427, *P*=.03), and parents’ education (beta=–0.528, standardized beta=–0.418, *P*=.007). All other variables were much less influential and were not significant at the 5% significance level.

**Table 16:** Opinions about minimum age to join an emergency response community (all values in years).

Population	N (valid^a^)	Average	Min	Max	SD	Median	95% CI
Parents	65	12.27	6	18	3.02	12	11.52-13.02
Children (attended)	23	13.15	6.5	20	3.44	12	11.63-14.67

^a^These data were missing in 8 parents’ questionnaires.

We used a linear regression to analyze the factors that influence children’s opinions about the minimum age to join an ERC. We used the following independent variables: child’s age, child’s adherence (number of days in the past week child had immediate access to AAI), parenting style, and child’s autonomy. A multicollinearity analysis did not reveal any evidence of multicollinearity. The model resulted in *R*^2^=0.602, and the model was very close to significance at the 5% significance level (*P*=.05). An analysis of standardized regression coefficients revealed that the most influential significant variables are monitoring parenting style (beta=4.668, standardized beta=0.952, *P*=.005) and protective parenting style (beta=–4.381, standardized beta=–0.888, *P*=.008). All other variables were much less influential and were not significant at the 5% significance level.

## Discussion

### Principal Findings

#### Parents’ Willingness to Join an Emergency Response Community

Parents’ willingness to join the community was very high, even for a convenience sample. In the following, we describe the main factors that influence parents’ willingness to join.

Parents’ willingness to join was negatively correlated with parents’ age and parents’ adherence. Parents’ age had a strong positive correlation with child’s age. Parents of younger children carried the AAIs for their children. These findings can be explained by the *free-rider effect* [56]: parents who carried an AAI for a young child, but had low adherence, wanted to join the ERC to get an additional layer of response in an emergency. Parents’ age was also negatively correlated with a protective parenting style, as this parenting style is associated with a need for additional safety measures [47] and joining an ERC can satisfy this need. Parents’ age was strongly positively correlated with child’s age, which was positively correlated with time since diagnosis and time since the last anaphylactic attack. Parents of newly diagnosed children had higher levels of parental anxiety [57,58], which led to higher willingness to join to get an additional layer of support.

Parents’ willingness to join an ERC was positively correlated with child’s adherence. Previous studies have found that parents’ psychological characteristics influence their child’s adherence [59]. A possible explanation for this finding is that parents’ characteristics, such as self-efficacy or parental warmth, which are known to be associated with higher adherence among children [60,61], are also associated with a higher probability of joining an ERC. Further research is required to verify this hypothesis.

We found that females were more likely to join the community than males. This can be explained both from a giving point of view (females participate more in volunteer activities than males [62] and have higher motivation to volunteer [63]) and from a taking point of view (mothers of children with a medical condition experience higher levels of parental anxiety than fathers [58,64] and are thus more likely than males to seek help for their children’s condition [65]).

#### Children’s Willingness to Join an Emergency Response Community

Children’s willingness to join the community was lower than that of their parents, but still high. Children’s willingness to join the community was positively correlated with age. This is a straightforward finding both from a giving point of view (age is positively correlated with volunteerism [66]) and from a taking point of view (age is positively correlated with help seeking [67]). Children’s willingness to join the community was also negatively correlated with children’s emotional autonomy. The latter correlation may be more of a methodological issue, as it may be related to the specific questions in the emotional autonomy subscale of the AAQ. For example, “I adapt myself to what other people want” (from question 7 of the AAQ) might be implicitly associated with lack of emotional autonomy in the context of joining an ERC.

#### Parents’ Willingness to Enroll Their Children in an Emergency Response Community

About half of the parents expressed willingness that their children join an ERC. Both the logistic regression and the classification tree identified that being the parent responsible to carry an AAI for his child and parent's adherence as significant factors negatively correlated with parent's willingness to enroll their child in an ERC. It seems that parents who are not able to provide their children with an AAI in the event of an anaphylactic attack want to enroll their children to provide them with an additional layer of support in an emergency.

Child’s age was positively correlated with parents’ willingness to enroll their children in the community. From a taking point of view, this can be explained by the transition from a protective parenting style, which is more common in parents of younger children, to a monitoring parenting style, which prevails as children grow older [68]. While the protective parenting style is characterized by the parents’ attempts to protect their child by themselves, the monitoring parenting style is characterized by the parents’ provision of tools to enable the child to cope by himself. Thus, parents of older children, especially those who begin to go out on their own, are more likely to view enrolling their child in an ERC as another tool that he can use in case of an emergency. From a giving point of view, age is positively correlated with volunteerism [66] and is also positively correlated with the ability to help another patient in an emergency, such as to provide him with cardiopulmonary resuscitation [69]. The negative correlation between a history of anaphylactic attacks in the past and parents’ willingness to enroll their children can be explained by the parents’ concerns that children who use their AAI to help another patient will remain unprotected until they get a replacement [42].

The 2 lower levels of the classification tree reveal another interesting effect: whereas parents of a young child want to enroll him if he has less than perfect adherence, parents of an older child want to enroll him only if he carries an AAI at least 3 days a week. The former finding can be explained by the *free-rider effect* and *taking* behavior among parents of younger children, who understand that their children probably would not be able to provide help to others. The latter finding appears to indicate *giving* behavior among parents of older children, who want to make sure that if their children join, they have a reasonable chance of helping others when called to action.

Cluster analysis with 2 clusters revealed 2 subgroups of 32 and 41 parents. The smaller group is characterized by younger parents, younger children, shorter time since diagnosis, shorter time since last attack, lower percentage of children who carry an AAI, higher percentage of parents who carry an AAI for their children, higher adherence among parents, lower adherence among children, and lower willingness to enroll child in an ERC. Cluster analysis with 3 clusters revealed 3 subgroups:

- Cluster 2: 14 parents to very young children. In this group, all parents carry an AAI for their children, and none of children carries the AAI for himself. Parents' adherence is high when compared with other clusters, and the willingness to enroll child in the community is very low.
- Cluster 1: 17 parents to adolescents. In this group, only 1 parent carries an AAI for his child, and all children carry an AAI for themselves. Adherence among children is the highest when compared with other clusters, and the willingness to enroll child in the community is very high.
- Cluster 0: 42 parents form the third cluster, which is in the middle between the 2 aforementioned clusters. Almost all children already carry an AAI for themselves, but many parents continue to carry an AAI. The adherence is a little bit lower than in cluster 1, but still high. The willingness to enroll a child in the community falls near the midpoint between clusters 1 and 2.

These results are consistent with previously described results obtained by other techniques. Parents’ willingness to enroll their child in the community is positively correlated with child's age and child's adherence.

#### Minimum Age to Join an Emergency Response Community

The minimum age for a child to join an ERC is between the ages of 12 and 13 years in the opinion of both parents and children. This can be explained by the fact that the study was performed in Israel where the ages of 12 years for girls and 13 years for boys are commonly considered the years when a child comes of age. Another possible explanation is that the age of 12 to 13 years is known to be the cutoff for cognitive development [70,71].

The parents’ opinion about the minimum age for a child to join an ERC was positively correlated with the age of the parents’ own child. This finding may be related to the well-known cognitive bias of *anchoring* [72], suggesting that parents rely on their own child’s age as an *anchor* when making decisions about the minimum age to join an ERC.

The parents’ opinion about the minimum age for a child to join an ERC was negatively correlated with the protective parenting style and positively correlated with the monitoring parenting style. These findings can be explained by the desire of parents with a higher protective parenting style to provide their children with an additional layer of support as soon as possible, and the desire of parents with a higher monitoring parenting style to provide their children with tools to cope by themselves when they become more independent.

The parents’ opinion about the minimum age for a child to join an ERC was negatively correlated with the parents’ education, that is, the more the parent is educated, the higher the likelihood she will allow her child to join an ERC at a younger age. Such association may be related to the relationship between the level of education and the exposure to updated technological solutions for treating medical conditions [73].

The children’s opinion about the minimum age for a child to join an ERC was negatively correlated with the protective parenting style and positively correlated with the monitoring parenting style. These findings are consistent with the findings about the parents’ opinion presented above. Children who have protective parents are prone to anxiety [74] and thus need the additional protection of joining an ERC as soon as possible, whereas children who have monitoring parents view an ERC as a tool that will help them to cope by themselves, more so as they grow older [75].

### Limitations

Our research used a convenience sample of highly motivated parents of children with food allergies, namely, those parents who decided to attend the annual meeting of a nonprofit organization for patients with food allergies (these attendees represent about 5% of the total membership of the organization).

Our study was limited to a single emergency condition: anaphylaxis. Most of the participants in our study were parents of patients, but not patients themselves. Our study was conducted in a single country: Israel. Cultural differences could lead to different results in different countries [76].

### Comparison With Prior Work

We observed high adherence levels among parents: 45 out of 50 parents (90%) reported that they always carry an AAI, 37 out of 52 parents (71%) reported that they had immediate access to an AAI every day in the past week, and 22 out of 52 parents (42%) had an AAI when filling out the questionnaire. Adherence was also high among children: 43 out of 57 parents (75%) reported that their children always carried an AAI, 51 out of 57 parents (89%) reported that their children had immediate access to AAI every day in the past week, and 53 out of 57 parents (93%) reported that the child had an AAI with him at the conference. These adherence levels are higher than those reported in 2 previous studies that found that 30% [77] and 26% to 45% [78] of patients carried an AAI at all times. However, these differences are not surprising among patients who decided to attend a conference and can thus be considered more motivated than those who stayed at home.

Comparison with the recent study by Shaker et al [42] is very instructive, given the fact that it also targeted parents of allergic children. The research question of Shaker et al was about sharing an AAI, and our research question was about joining an ERC, making our works complementary. Our findings that there are no significant differences in the willingness to join an ERC either by parents’ gender or by child’s gender or by parents’ education are consistent with the results of Shaker et al. They also found that a history of anaphylactic attacks was associated with a slightly higher willingness to share an AAI (79% vs 76%), but this finding was not statistically significant. In our study, the number of anaphylactic attacks in the past did not influence the willingness of parents to join an ERC, but it did negatively affect the willingness of parents to enroll their children in an ERC.

Shaker et al [52] reported that parents expressed concern about “leaving their own child without an AAI” and about the replacement cost of an AAI. It is therefore noteworthy that according to the EPIMADA protocol, an ambulance arriving on scene is required to provide a replacement AAI to the community member who responded to the emergency by giving his own AAI to the patient in distress. In other words, the EPIMADA protocol should alleviate the concerns identified by Shaker et al [42].

EPIMADA is a regulated community, and in every event, the volunteers are provided with guidance by phone from experienced EMS dispatchers. This approach can thus alleviate concerns about “harming the patient if it is not a real allergic reaction” that were also identified by Shaker et al [42].

### Conclusions

Our results provide strong support for the creation of ERCs for allergy patients. The willingness to join the community was found to be very high.

We studied a wide range of variables that can affect the willingness to join an ERC and described those variables that we found to be significant in our field study. Our findings can be of interest both to researchers of smartphone-based emergency response communities and to EMS administrators and policy makers who are considering establishing an ERC.

We note that mediation of an ERC by EMSs has the potential to solve several problems:

- An ambulance that arrives to the scene can replace the AAI of the community member who responded to the event by administering his own AAI to the patient in distress. This approach can alleviate the concerns about “leaving oneself or one’s child without an AAI” and those about replacement costs and delays.
- EMS dispatchers can provide guidance by phone. This approach can alleviate the concerns about “harming the patient” and can improve responders’ chances of providing emergency assistance.

Future research needs to address the following issues:

- Rare use of the app may cause users to forget to install it on new phones. Retention strategies should be developed.
- It is unclear whether ERC membership incentivizes adherence. On the one hand, a community member may feel responsibility to be ready to respond at any time, which could raise his level of adherence. On the other hand, a free-rider effect may lower his level of adherence.
- In this research, we focus on the willingness to join an ERC. Willingness to respond to an event is a different decision and needs to be studied.

## Appendix

Multimedia Appendix 1 Questionnaire for parents.

Multimedia Appendix 2 Questionnaire for children.

## Abbreviations

AAI: automatic adrenaline injector
AAQ: Adolescent Autonomy Questionnaire
ANOVA: analysis of variance
ARCS: Adult Responses to Children’s Symptoms
EMS: emergency medical service
ERC: emergency response community
ICC: intraclass correlation
MDA: Magen David Adom—the Israeli national EMS
OR: odds ratio
PCA: principal component analysis
