# Supplementary material for: Childrens' and Parents' Willingness to Join a Smartphone-Based Emergency Response Community for Anaphylaxis: Survey
Source: JMIR Mhealth Uhealth. 2019 Aug 27;7(8):e13892. doi: 10.2196/13892 (PMC6734855; doi:10.2196/13892)
Supplement: Multimedia Appendix 1 [file mhealth_v7i8e13892_app1.pdf]

Questionnaire for parents
Willingness to join the community for allergic people in emergencies
(translated from Hebrew)

Community for allergic people in emergencies - description

Anyone suffering from life-threatening allergies knows that the epinephrine injector that one carries, can save their life during an acute allergic reaction. Unfortunately, many patients do not carry the injector at all times, exposing themselves to risk.

The Social Network for Allergic People "Community for Allergic People in Emergencies" is a location-based application for smartphones developed in cooperation between Bar Ilan University and MDA.

The "Community for Allergic People in Emergencies" allows its members to give their personal epinephrine injector to another member who is nearby and is suffering from anaphylactic shock and does not have their own injector. Every delivery of emergency medicine between the members of the network is monitored and approved by MDA, thus enabling an immediate life-saving response for allergic people in an emergency situation, until the completion of the treatment by a medical team and sending an announcement about the end of the event to all members of the community nearby. MDA will immediately provide a replacement for an injector to the person who delivered his/her injector during an emergency.

It is important to clarify that members will not bear any legal or medical responsibility when participating in an emergency activity of the community.

The purpose of the questionnaire

The purpose of the questionnaire is to study the various properties that influence the decision to join the community for allergic people in emergencies.

The questionnaire is completely anonymous. The raw data will not be published. The study was approved by the Bar-Ilan University Institutional Review Board and by the MDA Research Committee.

Filling out the questionnaire is not mandatory and does not constitute a condition for participating in the conference.

Anyone who fills out the questionnaire in full and gives it to the research team, will receive a ticket for a lottery at the end of the conference.

Guidelines for filling out the questionnaire

Please fill out the questionnaire in full.

Please make sure to provide accurate answers – research based on your answers will be used to make important decisions.

Thank you for your cooperation!

Personal data - parent

Age (years)	____	Gender (circle)	male/female
Years of education	____	Level of religious observance (circle)	Secular / religious / ultra-orthodox
Number of children	____	Marital status (circle)	Single/Married/Divorced/Widowed

Allergy status - parent

Does the parent suffer from allergy?	Yes/No	Year of diagnosis	____
**Parent** - number of anaphylaxis attacks in the past	_____	Last attack – year	____
Is the parent **required** to carry an epinephrine injector <u>for themselves</u>?	Yes/No	First prescription – year	____
Does the parent undergo an allergy vaccination?	Yes/No	Months since the beginning of treatment	____
Does the parent **make sure** to carry an epinephrine injector for <u>themselves</u>?	Never/Seldom/Often/Always		

Personal data – child

Age (years)	_____	Gender (circle)	male/female

Allergy status - child

Does the child suffer from allergy	Yes/No	Year of diagnosis	____
**Child** - number of anaphylaxis attacks in the past	_____	Last attack – year	____
Does the child need an epinephrine injector available at all times?	Yes/No	First prescription – year	____
Does the child undergo an allergy vaccination?	Yes/No	Months since the beginning of treatment	____
Who carries the epinephrine injector for the child?	the child himself/the parent/both		
Does **the parent make sure** to carry the epinephrine injector <u>for the child</u>?	Never/Seldom/Often/Always		
Does **the child make sure** to carry the epinephrine injector for <u>themselves</u>?	Never/Seldom/Often/Always		

The community for allergic people in emergencies

Do you (parent) intend to join the community for allergic people in emergencies?	Yes/No
Do you intend to enroll your child in the community for allergic people in emergencies (as an independent user with a mobile phone with the app)?	Yes/No

What is the probability that you will join the community for allergic people in emergencies?	6	5	4	3	2	1	0
	Very likely						Very unlikely

What is the probability that you will enroll your child in the community for allergic people in emergencies (as an independent user with a mobile phone with the app)?	6	5	4	3	2	1	0
	Very likely						Very unlikely

In your opinion - what is the minimum age to enroll a child in the community for allergic people in emergencies (as an independent user with a mobile phone with the app) - provide age in years	________________

Note to questionnaire translation: In the Hebrew version all questions above this sentence fit a single page

Accessibility to Epipen questionnaire (*)

Question	Answer - circle						
Is there currently in your (parent) possession an epipen injector?	Yes/No						
Is there currently in your child's possession an epipen injector?	Yes/No						
How many days of the last week did you (the parent) have immediate access to the epipen injector throughout the day?	1	2	3	4	5	6	7
How many days of the last week did your child have immediate access to the epipen injector throughout the day?	1	2	3	4	5	6	7

(*) *Note to questionnaire translation:* In Israel, Epipen® is the only AAI available in the market and this brand name became a widely used synonym to AAI.

What happens when your child is ill?

The following questions relate to what you do when your child is ill (abdominal pain, headache). For each question, choose one of the following answers:

Never	this means **you** never do it
From time to time	this means that **you** do it only occasionally
Sometimes	this means **you** are doing it part of the time
Often	this means **you** usually do it
Always	this means **you** always do it

When your child is ill, how often do you…

	Always	Often	Sometimes	From time to time	Never
**1. Ask your child what you can do to help**	4	3	2	1	0
**2. Express irritation or frustration with your child**	4	3	2	1	0
**3. Do your child's chores or pick up his things instead of making him do it**	4	3	2	1	0
**4. Talk to your child about something else to take his mind off that**	4	3	2	1	0
**5. Give your child some medicine**	4	3	2	1	0
**6. Reassure your child that he is going to be ok**	4	3	2	1	0
**7. Get your child something to eat or drink**	4	3	2	1	0
**8. Bring your child special treats or little gifts**	4	3	2	1	0
**9. Try not to pay attention to your child**	4	3	2	1	0
**10. Ask your child questions about how he feels**	4	3	2	1	0
**11. Let your child stay home from school**	4	3	2	1	0
**12. Encourage your child to do something he enjoys (like watching TV to playing a game)**	4	3	2	1	0
**13. Tell your child that he does not have to finish all of her homework**	4	3	2	1	0

	Always	Often	Sometimes	From time to time	Never
14. Tell your child there is nothing you can do about it	4	3	2	1	0
15. Give your child special privileges	4	3	2	1	0
16. Stay home from work, come home early (or stay home instead of going out)	4	3	2	1	0
17. Tell others in the family not to bother your child or be especially nice	4	3	2	1	0
18. Tell your child not to make such a fuss about it	4	3	2	1	0
19. Pay more attention than usual to your child	4	3	2	1	0
20. Let your child to sleep in a special place (like in your room or on the couch	4	3	2	1	0
21. Tell your child that he needs to learn to be stronger	4	3	2	1	0
22. Let your child sleep later than usual in the morning	4	3	2	1	0
23. Keep your child inside the house	4	3	2	1	0
24. Try to involve your child in some activity	4	3	2	1	0
25. Spend more time than usual with your child	4	3	2	1	0
26. Try to make your child as comfortable as possible	4	3	2	1	0
27. Tell your child you still expect him to do chores or collect his things scattered around the house	4	3	2	1	0
28. Check on your child to see how he is doing	4	3	2	1	0
29. Call the doctor or take your child to the doctor	4	3	2	1	0
