# Supplementary material for: Childrens' and Parents' Willingness to Join a Smartphone-Based Emergency Response Community for Anaphylaxis: Survey
Source: JMIR Mhealth Uhealth. 2019 Aug 27;7(8):e13892. doi: 10.2196/13892 (PMC6734855; doi:10.2196/13892)
Supplement: Multimedia Appendix 2 [file mhealth_v7i8e13892_app2.pdf]

# Questionnaire for children
# Willingness to join the community for allergic people in emergencies
# (translated from Hebrew)

## Community for allergic people in emergencies - description

Anyone suffering from life-threatening allergies knows that the epinephrine injector that one carries, can save their life during an acute allergic reaction. Unfortunately, many patients do not carry the injector at all times, exposing themselves to risk.

The Social Network for Allergic People "Community for Allergic People in Emergencies" is a location-based application for smartphones developed in cooperation between Bar Ilan University and MDA.

The "Community for Allergic People in Emergencies" allows its members to give their personal epinephrine injector to another member who is nearby and is suffering from anaphylactic shock and does not have their own injector. Every delivery of emergency medicine between the members of the network is monitored and approved by MDA, thus enabling an immediate life-saving response for allergic people in an emergency situation, until the completion of the treatment by a medical team and sending an announcement about the end of the event to all members of the community nearby. MDA will immediately provide a replacement for an injector to the person who delivered his/her injector during an emergency.

It is important to clarify that members will not bear any legal or medical responsibility when participating in an emergency activity of the community.

### The purpose of the questionnaire

The purpose of the questionnaire is to study the various properties that influence the decision to join the community for allergic people in emergencies.

The questionnaire is completely anonymous. The raw data will not be published. The study was approved by the Bar-Ilan University Institutional Review Board and by the MDA Research Committee.

Filling out the questionnaire is not mandatory and does not constitute a condition for participating in the conference.

Anyone who fills out the questionnaire in full and gives it to the research team, will receive a ticket for a lottery at the end of the conference.

### Guidelines for filling out the questionnaire

Please fill out the questionnaire in full.

Please make sure to provide accurate answers – research based on your answers will be used to make important decisions.

**Thank you for your cooperation!**

**Willingness to join the community for allergic people in emergencies**

| | |
|---|---|
| If your parents let you, do you intend to join the community (as an independent user with a mobile phone with the app)? | Yes/No |

| | 6 | 5 | 4 | 3 | 2 | 1 | 0 |
|---|---|---|---|---|---|---|---|
| If your parents let you, what is the probability that you will join the community for allergic people in emergencies (as an independent user with a mobile phone with the app)? | Very likely | | | | | | Very unlikely |

| | |
|---|---|
| In your opinion - what is the minimum age to enroll a child in the community for allergic people in emergencies (as an independent user with a mobile phone with the app) - provide age in years | ________________ |

<u>**Accessibility to Epipen questionnaire (*)**</u>

| Question | Answer - circle | | | | | | |
|---|---|---|---|---|---|---|---|
| Is there currently in your possession an epipen injector? | Yes/No | | | | | | |
| How many days of the last week did you have immediate access to the epipen injector throughout the day? | 1 | 2 | 3 | 4 | 5 | 6 | 7 |

(*) *Note to questionnaire translation:* In Israel, Epipen® is the only AAI available in the market and this brand name became a widely used synonym to AAI.

יהל העמותה לאלרגיות מזון

אוניברסיטת בר-אילן

MAGEN DAVID ADOM IN ISRAEL מגן דוד אדום בישראל זה עניין של חיים

# Questionnaire for children (above 8 years old)

## FAIM

- The following four questions are about the chance that you think you have of something happening to you because of your food allergy.
- Choose one of the answers.
- This is followed by two more questions about your food allergy.
- Answer every question by putting an 'x' in the box next to proper answer.
- Answer according to the following rating:
    0. **Never (0% chance)**
    1. **Very small chance**
    2. **Small chance**
    3. **Fair chance**
    4. **Great chance**
    5. **Very big chance**
    6. **Always (100% chance)**

| How big do you think the chance is that you… | Never → 0 | 1 | 2 | 3 | 4 | 5 | Always 6 |
|---|---|---|---|---|---|---|---|
| 1. Will accidentally eat something to which you are allergic? | ☐ | ☐ | ☐ | ☐ | ☐ | ☐ | ☐ |
| 2. Will have a severe reaction if you accidentally eat something to which you are allergic? | ☐ | ☐ | ☐ | ☐ | ☐ | ☐ | ☐ |
| 3. Will die if you accidentally eat something to which you are allergic? | ☐ | ☐ | ☐ | ☐ | ☐ | ☐ | ☐ |
| 4. Can <u>not</u> effectively deal with an allergic reaction, should you accidentally eat something to which you are allergic? | ☐ | ☐ | ☐ | ☐ | ☐ | ☐ | ☐ |

*Note to questionnaire translation: In the Hebrew version, question below this sentence were on a separate page*

| How many foods are you unable to eat because of your food allergy? | |
|---|---|
| ☐ almost none | ☐ so little I don't actually notice it |
| ☐ very few | ☐ very little |
| ☐ a few | ☐ a little |
| ☐ some | ☐ moderately |
| ☐ many | ☐ a good deal |
| ☐ very many | ☐ a great deal |
| ☐ almost all | ☐ a very great deal |

**Here are a few sentences. For each sentence, choose one of the following answers:**

| | |
|---|---|
| A very bad description of myself | When the statement does not describe you at all |
| A bad description of myself | When the statement does not describe you |
| An average description of myself | When the statement describes you sometimes |
| A good description of myself | When the statement describes you |
| A very good description of myself | When the statement describes you very much |

| | | A very bad description of myself | A bad description of myself | An average description of myself | A good description of myself | A very good description of myself |
|---|---|---|---|---|---|---|
| **1** | I find it difficult to decide what I want | 1 | 2 | 3 | 4 | 5 |
| **2** | I can easily choose | 1 | 2 | 3 | 4 | 5 |
| **3** | I often do not know what to think | 1 | 2 | 3 | 4 | 5 |
| **4** | When people ask me what I want, I immediately know the answer | 1 | 2 | 3 | 4 | 5 |
| **5** | I often hesitate about what to do | 1 | 2 | 3 | 4 | 5 |
| **6** | When I act against the wishes of others, I become stressed | 1 | 2 | 3 | 4 | 5 |
| **7** | I have a strong tendency to obey the will of others | 1 | 2 | 3 | 4 | 5 |
| **8** | When I disagree with others, I tell them | 1 | 2 | 3 | 4 | 5 |
| **9** | I often agree with others, even if I'm not sure | 1 | 2 | 3 | 4 | 5 |
| **10** | I often change my mind after listening to others | 1 | 2 | 3 | 4 | 5 |
| **11** | I'm going straight on my goal | 1 | 2 | 3 | 4 | 5 |
| **12** | I find it difficult to start a new activity on my own | 1 | 2 | 3 | 4 | 5 |
| **13** | I can easily start with new commitments | 1 | 2 | 3 | 4 | 5 |
| **14** | I am an adventurous child | 1 | 2 | 3 | 4 | 5 |
| **15** | I quickly feel at ease in new situations | 1 | 2 | 3 | 4 | 5 |

יהל העמותה לאלרגיות מזון

אוניברסיטת בר-אילן

MAGEN DAVID ADOM IN ISRAEL מגן דוד אדום בישראל זה עניין של חיים

# What happens when you are ill?

The following questions relate to what your parents do when you are ill (abdominal pain, headache). For each question, choose one of the following answers:

| | |
|---|---|
| Never | this means they **never** do it |
| From time to time | this means that they do it **only occasionally** |
| Sometimes | this means they are doing it **part of the time** |
| Often | this means they **usually** do it |
| Always | this means they **always** do it |

**When you are ill, how often do your parents…**

| | Never | From time to time | Sometimes | Often | Always |
|---|---|---|---|---|---|
| **1. Ask you what they can do to help?** | 0 | 1 | 2 | 3 | 4 |
| **2. Express irritation or frustration with you?** | 0 | 1 | 2 | 3 | 4 |
| **3. Do your chores or pick up your things instead of making you do it?** | 0 | 1 | 2 | 3 | 4 |
| **4. Talk to you about something else to take your mind off that?** | 0 | 1 | 2 | 3 | 4 |
| **5. Give you some medicine?** | 0 | 1 | 2 | 3 | 4 |
| **6. Reassure you that you are going to be ok?** | 0 | 1 | 2 | 3 | 4 |
| **7. Get you something to eat or drink?** | 0 | 1 | 2 | 3 | 4 |
| **8. Bring you special treats or little gifts?** | 0 | 1 | 2 | 3 | 4 |
| **9. Try not to pay attention to you?** | 0 | 1 | 2 | 3 | 4 |
| **10. Ask you questions about how you feel?** | 0 | 1 | 2 | 3 | 4 |
| **11. Let you stay home from school?** | 0 | 1 | 2 | 3 | 4 |
| **12. Encourage you to do something you enjoy (like watching TV to playing a game)?** | 0 | 1 | 2 | 3 | 4 |
| **13. Tell you that you do not have to finish all of your homework?** | 0 | 1 | 2 | 3 | 4 |
| **14. Tell you there is nothing they can do about it?** | 0 | 1 | 2 | 3 | 4 |
| **15. Give you special privileges?** | 0 | 1 | 2 | 3 | 4 |
| **16. Stay home from work, come home early (or stay home instead of going out)?** | 0 | 1 | 2 | 3 | 4 |
| **17. Tell others in the family not to bother you or be especially nice?** | 0 | 1 | 2 | 3 | 4 |

| | Never | From time to time | Sometimes | Often | Always |
|---|---|---|---|---|---|
| **18. Tell you not to make such a fuss about it?** | 0 | 1 | 2 | 3 | 4 |
| **19. Pay more attention to you than usual?** | 0 | 1 | 2 | 3 | 4 |
| **20. Let you to sleep in a special place (like in their room or on the couch)?** | 0 | 1 | 2 | 3 | 4 |
| **21. Tell you that you need to learn to be stronger?** | 0 | 1 | 2 | 3 | 4 |
| **22. Let you child sleep later than usual in the morning?** | 0 | 1 | 2 | 3 | 4 |
| **23. Keep you inside the house?** | 0 | 1 | 2 | 3 | 4 |
| **24. Try to involve you in some activity?** | 0 | 1 | 2 | 3 | 4 |
| **25. Spend more time than usual with you?** | 0 | 1 | 2 | 3 | 4 |
| **26. Try to make you as comfortable as possible?** | 0 | 1 | 2 | 3 | 4 |
| **27. Tell you that they still expect you to do chores or collect your things scattered around the house?** | 0 | 1 | 2 | 3 | 4 |
| **28. Check on you to see how you are doing?** | 0 | 1 | 2 | 3 | 4 |
| **29. Call the doctor or take you to the doctor?** | 0 | 1 | 2 | 3 | 4 |
